# Rapid Single-Step Immunochromatographic Assay for *Angiostrongylus cantonensis* Specific Antigen Detection

**DOI:** 10.3390/pathogens12060762

**Published:** 2023-05-25

**Authors:** Praphathip Eamsobhana, Anchalee Tungtrongchitr, Darawan Wanachiwanawin, Sudarat Boonyong, Hoi-Sen Yong

**Affiliations:** 1 Department of Parasitology, Faculty of Medicine Siriraj Hospital, Mahidol University, Bangkok 10700, Thailand; darawan.wan@mahidol.ac.th (D.W.); sudarat.boo@mahidol.ac.th (S.B.); 2 Institute of Biological Sciences, Faculty of Science, Universiti Malaya, Kuala Lumpur 50603, Malaysia; yong@um.edu.my

**Keywords:** *Angiostrongylus cantonensis*, lateral flow assay, immunochromatographic test, antigen detection, 31-kDa antigen, active angiostrongyliasis

## Abstract

*Angiostrongylus cantonensis* is the major etiological nematode parasite causing eosinophilic meningitis and/or eosinophilic meningoencephalitis in humans. The rapid global spread of *Angiostrongylus cantonensis* and the emerging occurrence of the infection have exposed the shortcomings of traditional/conventional diagnostics. This has spurred efforts to develop faster, simpler and more scalable platforms that can be decentralized for point-of-need laboratory testing. By far, the point-of-care immunoassays such as the lateral flow assay (LFA) are the best-placed. In this work, a LFA in the form of an immunochromatographic test device (designated *Ac*AgQuick^Dx^), based on the detection of a circulating *Angiostrongylus cantonensis*-derived antigen, was established using anti-31 kDa *Angiostrongylus cantonensis* antibody as the capture reagent and anti-*Angiostrongylus cantonensis* polyclonal antibody as the indicator reagent. The *Ac*AgQuick^Dx^ was evaluated for its diagnostic potential with a total of 20 cerebrospinal fluids (CSF) and 105 serum samples from patients with angiostrongyliasis and other clinically related parasitic diseases, as well as serum samples from normal healthy subjects. Three of the ten CSF samples from serologically confirmed angiostrongyliasis cases and two of the five suspected cases with negative anti-*Angiostrongylus cantonensis* antibodies showed a positive *Ac*AgQuick^Dx^ reaction. Likewise, the *Ac*AgQuick^Dx^ was able to detect *Angiostrongylus cantonensis* specific antigens in four serum samples of the 27 serologically confirmed angiostrongyliasis cases. No positive reaction by *Ac*AgQuick^Dx^ was observed in any of the CSF (*n* = 5) and serum (*n* = 43) samples with other parasitic infections, or the normal healthy controls (*n* = 35). The *Ac*AgQuick^Dx^ enabled the rapid detection of active/acute *Angiostrongylus cantonensis* infection. It is easy to use, can be transported at room temperature and does not require refrigeration for long-term stability over a wide range of climate. It can supplement existing diagnostic tests for neuroangiostrongyliasis under clinical or field environments, particularly in remote and resource-poor areas.

## 1. Introduction

*Angiostrongylus cantonensis*, also known as rat lungworm, is an angiostrongylid nematode parasite that typically resides in the pulmonary arteries and right ventricle of rodents. It is the commonest etiological parasite causing eosinophilic meningitis and/or eosinophilic meningoencephalitis in humans [1,2]. This neurotropic parasite is of increasing public health importance as its geographic distribution now covers a wide part of the world, and new locality records continue to be reported [2,3,4,5,6]. 

*Angiostrongylus cantonensis* has a relatively simple heteroxenous life cycle, typically involving a definitive rodent host and a mollusk intermediate host, but it may also use various paratenic hosts [2,7]. Humans are an accidental host, acquiring the infection via the ingestion of raw or poorly cooked snail meat and a variety of paratenic hosts which harbor the third-stage larvae, or green vegetables contaminated with the infective larvae [1,2,3,6,8]. The most frequently reported symptoms are headache, neck stiffness, paresthesia, fever, visual disturbances, vomiting and nausea [1,2,3,4,5,9]. The migration of the larvae to the brain tissue causes serious central nervous system (CNS) damage, which can result in coma and death of the patient [10,11].

The definitive diagnosis for human neural angiostrongyliasis is based on the detection of immature worms in the cerebrospinal fluid (CSF) or from the eye of infected patients, but such findings are of rare occurrence [9]. The disease is presumptively diagnosed based on presenting symptoms, medical histories, eosinophilic pleocytosis in the CSF, and immunological and/or molecular markers [2,3,4,12].

Antibody-based immunodiagnostic tests using purified antigens for neuroangiostrongyliasis have been available for decades. Most of the work has centered on the *Angiostrongylus cantonensis* 31-kDa glycoprotein antigen [13,14], 29-kDa antigen [15], and 32-kDa protein [16]. The 31-kDa glycoprotein antigen of *Angiostrongylus cantonensis* has mostly been used in traditional immunoblotting as a diagnostic marker for differential diagnosis in human angiostrongyliasis, with very high sensitivity and specificity [17,18,19]. On the other hand, the detection of circulating *Angiostrongylus cantonensis* antigens is an alternative immunodiagnostic test to identify the active/acute/early stages of neuroangiostrongyliasis, and it is very much required for the patient management/treatment. 

Furthermore, a molecular approach that targets gene sequences of *Angiostrongylus cantonensis* can also assist in an early etiologic diagnosis. Various DNA-based diagnostic techniques that rely on polymerase chain reaction (PCR) to amplify and detect specific *Angiostrongylus cantonensis* DNA molecules have been successfully applied to detect *Angiostrongylus cantonensis* DNA in cerebrospinal fluid (CSF)/clinical specimens [20,21,22].

Because parasitological/definitive diagnosis is rarely achieved, immunological and/or molecular diagnostic methods for the detection of *Angiostrongylus cantonensis* antibodies/antigens and/or nucleic acids have become widely accepted as the most appropriate diagnostic approach to support clinical diagnosis [2,12,23]. Nevertheless, the utilization of such tests is time-consuming, needs highly sophisticated, high-cost laboratory equipment and continuous electricity supply and is not suitable under clinical or field conditions in endemic regions [22].

The rapid global spread of *Angiostrongylus cantonensis* and the emerging occurrence of the infection have exposed the shortcomings of traditional/conventional diagnostics. This has spurred efforts to develop faster, simpler and more scalable platforms that can be decentralized for performing the on-site detection of *Angiostrongylus cantonensis* infection in order to allow prompt treatment decisions. At present, point-of-care immunoassays, such as the flow-through/lateral flow assay, are the best placed.

The LFA has recently attracted considerable interest because of its long shelf life and the fact that refrigeration is not required for storage. It is very well adapted for application in harsh field environments, and in remote regions. As such, an LFA in the form of an immunochromatographic test device (*Ac*AgQuick^Dx^) based on the detection of a circulating *Angiostrongylus cantonensis*-derived antigen was established, using an anti-31 kDa *Angiostrongylus cantonensis* antibody line on the membrane strip as the capture reagent and anti-*Angiostrongylus cantonensis* polyclonal antibody conjugated to colloidal gold as the indicator reagent. The *Ac*AgQuick^Dx^ was initially evaluated for its diagnostic potential in this study.

## 2. Materials and Methods

### 2.1. Clinical Samples

A set of 20 individual CSF samples submitted to the Parasitology Laboratory, Department of Parasitology, Faculty of Medicine Siriraj Hospital, Bangkok, Thailand, for routine antibody testing (ELISA and/or immunoblot) of tissue-invading parasites, i.e., *Gnathostoma spinigerum*, *Angiostrongylus cantonensis* and *Taenia solium* metacestodes, were used for the present assessment. These CSF specimens were from clinically diagnosed cases with positive immunoblot tests for the presence of a 31-kDa band specific for *Angiostrongylus cantonensis* (*n* = 10; designated as CSF1–10) and clinically suspected cases with negative immunoblot tests for *Angiostrongylus cantonensis* infection (*n* = 5; CSF11–15), as well as CSF samples (representing other clinically related parasitic infections) with positive immunoblot tests showing a 24-kDa band specific for *Gnathostoma spinigerum* (*n* = 2; CSF16–17) and with ELISA-positive cases of *Taenia solium* neurocysticercosis (*n* = 3; CSF18–20). The CSF specimens were kept at −70 °C after the initial routine immunological investigations. 

In addition, a total of 105 reference sera stock from the Parasitology Department of the Faculty of Medicine Siriraj Hospital, were also used for evaluation testing. Twenty-seven samples were from clinically diagnosed patients with detectable *Angiostrongylus cantonensis*-specific antibody in immunoblotting. The remaining 43 serum samples were from patients with other parasitic diseases, i.e., gnathostomiasis (*n* = 13), toxocariasis (*n* = 2), trichinellosis (*n* = 2), hookworm infection (*n* = 4), filariasis (*n* = 5), cysticercosis (*n* = 9), paragonimiasis (*n* = 2), opisthorchiasis (*n* = 3) and malaria (*n* = 3). These infections had been diagnosed using parasitological or serological methods. Additionally, 35 serum samples from normal healthy subjects (whose stool samples were without any intestinal parasitic infection) were included for testing. 

All the 105 patient sera and 20 cerebrospinal fluids were collected from the leftover clinical samples stored separately at −70 °C. The same sets of archived CSF (*n* = 20) and serum (*n* = 97) samples had been tested with *Ac*DIGFA^Ag^ to detect the 31-kDa specific antigen of *Angiostrongylus cantonensis* [24]. Additionally, 8 serum specimens used in this study were from cases with positive anti-*Angiostrongylus cantonensis* antibodies detected using an immunoblot test. They were retrieved and re-tested using *Ac*AgQuick^Dx^ to confirm the presence of a 31-kDa *Angiostrongylus cantonensis* antigen. The use of stored leftover clinical CSF or serum samples for this study was approved by the Director of Siriraj Hospital, Faculty of Medicine Siriraj Hospital, Mahidol University.

### 2.2. Production and Purification of Polyclonal Antibodies

Procedures to produce rabbit immune sera against crude somatic extracts and purified 31-kDa glycoprotein of *Angiostrongylus cantonensis* were the methods previously described [24,25,26,27]. The rabbit antisera used in this study were those previously produced and stored in small aliquots at −70 °C. Anti-*Angiostrongylus cantonensis* and anti-31 kDa *Angiostrongylus cantonensis* immune sera collected 2 weeks after the last immunizing dose were used to establish the present *Ac*AgQuick^Dx^ device. From the previous immunoblot analysis, the crude *Angiostrongylus cantonensis* extracts were recognized using anti-*Angiostrongylus cantonensis* polyclonal antibody as multiple protein bands, including the 31-kDa antigenic band, whereas the anti-31 kDa antibody recognized a broad band with an approximate molecular mass of 31 kDa. The antisera were retrieved and purified using the Melon IgG Spin Purification Kit (Thermo Scientific, Waltham, MA, USA) according to the manufacturer’s instructions. After purification, the viability of the purified rabbit antibodies was confirmed to ensure the test performance. The purified anti-31 kDa *Angiostrongylus cantonensis* IgG was used as an antigen-capture antibody, while the purified anti-*Angiostrongylus cantonensis* IgG was used for colloidal gold-labelling as a detection agent.

### 2.3. Preparation of the Lateral Flow Test Device

The colloidal gold-labelled polyclonal antibody against *Angiostrongylus cantonensis* was prepared and the immunochromatographic test device (designated *Ac*AgQuick^Dx^) was then assembled as per the standard method by Serve Science Co., Ltd., Bangkok, Thailand. The lateral flow strip consisted of 4 components, i.e., the sample application pad, conjugate pad, antibody-immobilized nitrocellulose membrane and absorbent pad. In the detection zone/membrane, the purified anti-31 kDa *Angiostrongylus cantonensis* polyclonal antibody (1 mg/mL) was micro-sprayed (in a 1 mm wide line) at a flow rate of 0.1 µL per mm at the test line (T), and the goat anti-rabbit immunoglobublin-G (0.4 mg/mL) was sprayed at 0.1 µL per mm at the control line (C). The anti-*Angiostrongylus cantonensis* polyclonal antibody-coated colloidal gold probe was added to the conjugate pad as a detector reagent. The antibody-immobilized membrane and conjugate pad were dried overnight at 37 °C. The sample application pad, conjugate pad and absorbent pad were assembled with the antibody-immobilized nitrocellulose membrane on a laminated card by superposition/overlapping of the different pads, and was cut into strips (0.5 cm in width). Each test strip was housed in a protective plastic cassette, with a hole for sample application and a slot/window to display test results for interpretation, and then stored in a desiccated sealed aluminum foil-package at room temperature until used. The complete set of the *Ac*AgQuick^Dx^ kit consisted of an immunochromatographic cassette and chromatographic/chasing buffer.

### 2.4. Procedure of Lateral Flow Test Device

The test procedure was performed at room temperature. In the testing process, 25 µL of the test CSF/serum was applied slowly into the sample hole. After being completely absorbed, one drop (approximately 50 µL) of chromatographic/chasing buffer was added and allowed to be absorbed through the membrane to wash the excess colloidal gold-conjugated antibody/IgG. The result was interpreted within 15 min. The appearance of a red-colored band at the test (T) line and a red-colored band at the control (C) line indicated a positive result, whereas the absence of a red-colored band at the test (T) line and appearance of a red-colored band at the control (C) line indicated a negative result. The test was invalid when no red-colored bands appeared at either the T and C lines, or only one red-colored band appeared at the T line. All the parasite-infected patient CSF/serum samples and healthy control sera were tested twice with the *Ac*AgQuick^Dx^ device to confirm the reproducibility of the results. 

## 3. Results

**Figure 1:**
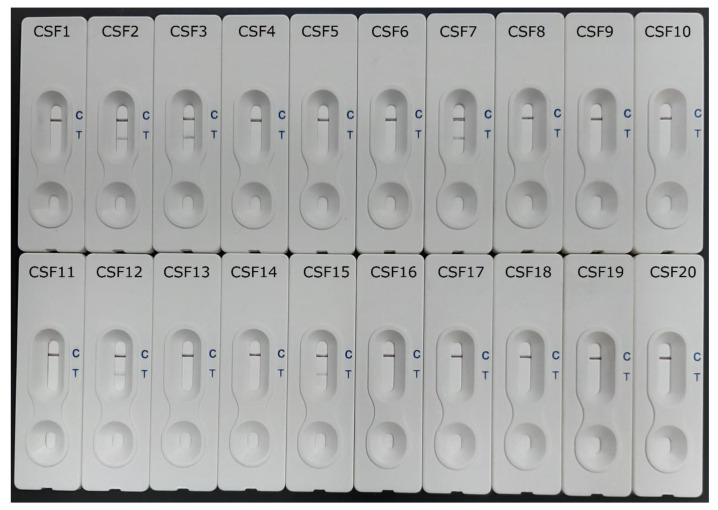
The *Ac*AgQuick^Dx^ detection results of 20 CSF samples from clinically diagnosed cases with positive immunoblot (antibody) test for angiostrongyliasis (CSF1–10), clinically suspected cases with negative immunoblot test (CSF11–15), and CSF samples with positive immunoblot (antibody test) for gnathostomiasis (CSF16–17), and with ELISA-positive cases of *Taenia solium* neurocysticercosis (CSF18–20). The appearance of a red band at the T line and a red band at the C line indicates a positive sample for the detection of the specific 31-kDa *Angiostrongylus cantonensis* antigen (CSF2, 3, 7, 12 and 15), whereas the absence of a red band at the T line and appearance of a red band at the C line indicates a negative sample (CSF1, 4, 5, 6, 8–11 and 13–14 and 16–20).

**Figure 2:**
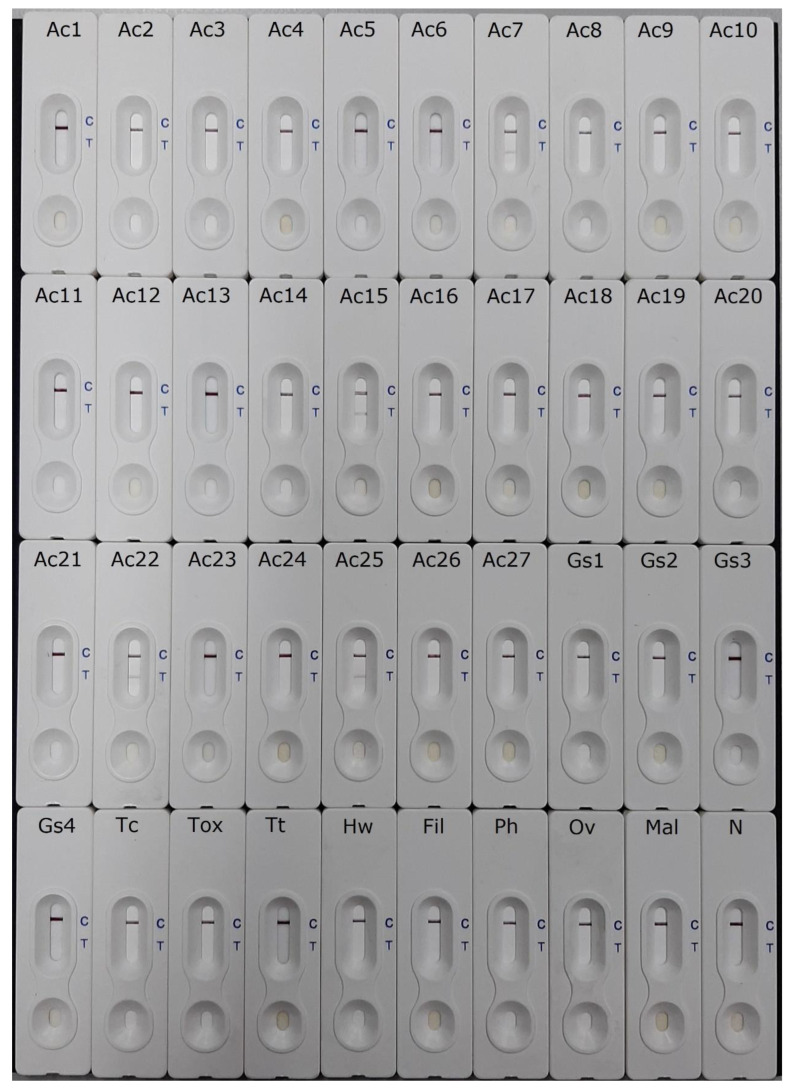
The *Ac*AgQuick^Dx^ test results of 27 serum samples from clinically diagnosed cases of angiostrongyliasis with positive immunoblot (antibody) test (Ac1–27), and the representative images of *Ac*AgQuick^Dx^ test evaluated using human sera with other heterologous parasitic infections (*n* = 43). Gs, gnathostomiasis; Tc, *Taenia solium* neurocysticercosis; Tox, toxocariasis; Tt, trichinellosis; Hw, hookworm infection; Fil, filariasis; Ph, paragonimiasis; Ov, opisthorchiasis; Mal, malaria; and N, normal healthy control (*n* = 35). The appearance of a red band at the T line and a red band at the C line indicates a positive result for detection of the specific 31-kDa antigen of *Angiostrongylus cantonensis* (Ac7, Ac15, Ac22 and Ac25). The absence of a red band at the T line and appearance of a red band at the C line indicates a negative test result (Ac1–6, Ac8–14, Ac16–21, Ac23–24, Ac26–27, Gs1–4, Tc, Tox, Tt, Hw, Fil, Ph, Ov, Mal and N).

In this work, of the 10 CSF samples from patients showing clinical criteria of eosinophilic meningitis and positive results for a 31-kDa *Angiostrongylus cantonensis*-specific immunoblot band, three (CSF2, 3 and 7) had a positive *Ac*AgQuick^Dx^ reaction, showing positive red bands (with different color intensities) at the test (T) region within 15 min. Likewise, two (CSF12 and CSF15) of the five CSF samples from cases with clinical features of infection, but which were negative for *Angiostrongylus cantonensis*-specific antibodies, also displayed a positive *Ac*AgQuick^Dx^ test (Figure 1). Within the set of 27 patient sera with serologically confirmed *Angiostrongylus cantonensis* infection, four (Ac-7, Ac-15, Ac-22 and Ac-25) showed a positive reaction via *Ac*AgQuick^Dx^, with visible pink bands at the T region (Figure 2). No positive *Ac*AgQuick^Dx^ reaction was observed in the other 23 sera with angiostrongyliasis. In cross-reactivity testing, all the 5 CSF samples and the 78 sera from gnathostomiasis (CSF = 2; serum = 13), toxocariasis (serum = 2), filariasis (serum = 5), trichinellosis (serum = 2), hookworm infection (serum = 4), cysticercosis (CSF = 3; serum = 9), paragonimiasis (serum = 2), opisthorchiasis (serum = 3), and malaria (serum = 3), as well as the 35 normal control sera from parasite-free individuals, were all negative for *Ac*AgQuick^Dx^.

## 4. Discussion

In clinical practice, the delayed diagnosis of *Angiostrongylus* eosinophilic meningitis due to its atypical symptoms is an important problem in many hospitals because it can cause fatal outcomes. Despite the recent introduction of a powerful and sensitive molecular diagnostic tool (metagenomic next-generation sequencing) for the specific identification of *Angiostrongylus cantonensis* DNA sequences in clinical specimens [28,29], the diagnosis of active/acute neuroangiostrongyliasis remains a challenge in resource-limited settings because of high operating costs and the requirement for laboratory infrastructure [29]. Other rapid and cost-effective assays for the point-of-need diagnosis are still required to meet the demand of appropriate tests for use in resource-poor settings or remote endemic regions.

In the early stages of infection, diagnosis based on antibody-based methods may lack sensitivity, especially during acute illness because seroconversion may take several weeks [30]. Alternatively, the detection of antigens has the advantage of detecting the presence of active *Angiostrongylus cantonensis* infection and the level of the infective burden. To date, only a few diagnostic tests have been reported for detecting specific *Angiostrongylus cantonensis* antigen in clinical samples. A rapid lateral flow immunoassay (LFIA), based on two monoclonal antibodies (12D5C12, 21B7B11) for detecting the specific antigens of *Angiostrongylus cantonensis*, revealed very high specificity (100%) and high sensitivity (91.1%) [31]. More recently, a rapid, non-enzymatic, dot immunogold filtration assay (*Ac*DIGFA^Ag^) based on flow-through immunoassay, using purified antibodies against specific 31-kDa *Angiostrongylus cantonensis* antigen to detect a corresponding (specific) *Angiostrongylus cantonensis* antigen in the cerebrospinal fluid and serum samples from angiostrongyliasis patients, showed a diagnostic specificity of 100% [24].

The specific 31-kDa glycoprotein antigen of *Angiostrongylus cantonensis* has been suggested to be a potential immunological marker of early infection since this 31-kDa antigen is a component of the *Angiostrongylus cantonensis* excretory/secretory products [32]. This antigen has shown considerable promise as an immunodiagnostic target in an assay protocol based on the flow through/vertical flow principle [24]. However, the additional gold detector reagent for the test assay still needs refrigeration; as such, it is not completely applicable in the harsh field environment. As the relatively high concentration of the circulating 31-kDa antigen of *Angiostrongylus cantonensis* facilitates the development of other test platforms, this specific antigen was selected for application in a more field-friendly lateral flow assay. In addition, the gold-based, lateral flow immunochromatography approach to detect a specific 31-kDa glycoprotein of *Angiostrongylus cantonensis* (*Ac*AgQuick^Dx^ test) presented here was easily adapted from the previous method of *Ac*DIGFA^Ag^, used to detect the specific 31-kDa *Angiostrongylus cantonensis* antigen [24].

In this study, the evaluation of the diagnostic potential of the *Ac*AgQuick^Dx^, using a set of clinical samples from the reference stock, revealed 5 out of the 15 angiostrongyliasis CSF samples and 4 out of the 27 angiostrongyliasis serum samples to be positive, whereas none of the patient’s samples with other heterologous parasitic infections (*n* = 48) and normal controls (*n* = 35) gave positive reactions. The overall diagnostic sensitivity and specificity of *Ac*AgQuick^Dx^ in detecting the 31-kDa antigens of *Angiostrongylus cantonensis* were 21.43% and 100%, respectively. The low sensitivity/positivity of the *Ac*AgQuick^Dx^ test in this study could possibly relate to delays in the diagnosis of the suspected active angiostrongyliasis cases, as the sensitivity of the antigen test depends on the phase of the disease, and on the presence of circulating *Angiostrongylus cantonensis* specific antigens in the CSF and blood. In the chronic cases, the antibody levels are expected to be elevated due to the persistence of antigen stimulation. The formation of antigen–antibody complexes, by the circulating antigens and antibodies, may inhibit the detection of antigens in these clinical samples [33]. Additionally, in those patients with a low intensity of infection, the positive antigen levels may be very close to the cut-off value.

The same set of archived serum samples (*n* = 19) in the present study was tested earlier using *Ac*Quick^Dx^ (specific antibody detection) with 100% sensitivity and 98.72% specificity [34]. In this study, only two out of the nineteen samples showed positive antigen results based on the examination/detection of the specific *Angiostrongylus cantonensis* antigen on the consecutive angiostrongyliasis serum samples using *Ac*AgQuick^Dx^. On the other hand, two of the five CSF samples from clinically suspected cases (criteria of eosinophilic meningitis) with a negative immunoblot antibody test for *Angiostrongylus cantonensis* infection had positive antigen results using the present *Ac*AgQuick^Dx^ test. To overcome the limitations related to the early or late seroconversion phase of available clinical samples, the simultaneous use of both the rapid tests, *Ac*Quick^Dx^ (antibody detection) and *Ac*AgQuick^Dx^ (antigen detection), should be applied for accurate detection of neuroangiostrongyliasis.

In comparison to our present antigen-based immunochromatographic test, *Ac*AgQuick^Dx^, the earlier *Angiostrongylus cantonensis*-derived antigen detecting *Ac*DIGFA^Ag^ test, with the identical pair of *Angiostrongylus cantonensis-*specific antibodies, revealed a slightly better sensitivity for the consecutive CSF sample tested (CSF1–15; Ac1-Ac19). One of six positive CSF samples tested with *Ac*DIGFA^Ag^ (CSF5) was negative with *Ac*AgQuick^Dx^, whereas the two positive patient sera with *Ac*DIGFA^Ag^ gave concordant positive results with *Ac*AgQuick^Dx^. This is likely that flow-through is an immunoconcentration assay and thus allows the detection of less abundant antigens in the samples. 

Theoretically, in the antigen detection tests, monoclonal antibodies seem to be the better diagnostic reagents as they recognize a single epitope with high specificity. The *Ac*AgQuick^Dx^ in the present study showed 100% diagnostic specificity when tested with CSF/serum samples from patients with other clinically related parasitic infections. This was comparable with the earlier-developed *Ac*DIGFA^Ag^, which also demonstrated a specificity of 100% [24]. Nevertheless, additional clinical samples from cases with heterologous parasitic infections, including other infectious diseases and cancers that may cause eosinophil abnormality in CSF and peripheral blood, need to be performed for a more rigorous evaluation of the specificity.

Furthermore, only patients from a single geographical region (Thailand) were included in this study, limiting the certainty that these data could apply to other endemic countries/regions with different geographical strains of *Angiostrongylus cantonensis* [35]. Whether the *Ac*AgQuick^Dx^ will also perform equally well in other *Angiostrongylus cantonensis* endemic regions remains to be evaluated. In addition, the question of the *Ac*AgQuick^Dx^ test yielding qualitative or semi-quantitative results needs to be addressed. The future goals for improving our *Ac*AgQuick^Dx^ will be focused on identifying new signal amplification strategies as well as the quantitative system.

## 5. Conclusions

Overall, our *Ac*AgQuick^Dx^ test based on the detection of a circulating 31-kDa *Angiostrongylus cantonensis*-derived antigen is a fast, portable and easy-to-use test device that meets the needs of laboratory testing in a variety of healthcare settings. The test allows the rapid immunological diagnosis of *Angiostrongylus cantonensis* infection to enable immediate clinical management decisions to be made at or near the site of patient care (at the point of care settings). It can also be a good alternative for use in the initial screening of neuroagiostrongyliasis in large-scale investigations in the field, where sophisticated equipment is lacking.

## 6. Limitations of the Work

There was a limitation to the current study because the immunological testing was carried out retrospectively, using stored frozen CSF and sera. It is possible that the use of fresh clinical samples may increase the test sensitivity. 

## Data Availability

All the data applicable to this investigation are presented in this manuscript.
